# Ceramide activates lysosomal cathepsin B and cathepsin D to attenuate autophagy and induces ER stress to suppress myeloid-derived suppressor cells

**DOI:** 10.18632/oncotarget.13438

**Published:** 2016-11-17

**Authors:** Feiyan Liu, Xia Li, Chunwan Lu, Aiping Bai, Jacek Bielawski, Alicja Bielawska, Brendan Marshall, Patricia V. Schoenlein, Iryna O. Lebedyeva, Kebin Liu

**Affiliations:** ^1^ College of Life Sciences, Zhejiang University, Hangzhou, China; ^2^ Department of Biochemistry and Molecular Biology, Medical College of Georgia, Augusta, GA, USA; ^3^ Charlie Norwood VA Medical Center, Augusta, GA, USA; ^4^ Department of Biochemistry and Molecular Biology, Medical University of South Carolina, Charleston, SC, USA; ^5^ Department of Cell Biology and Anatomy, Medical College of Georgia, Augusta, GA, USA; ^6^ Department of Chemistry and Physics, Augusta University, Augusta, GA, USA; ^7^ Georgia Cancer Center, Augusta University, Augusta, GA, USA

**Keywords:** MDSCs, cathepsin, ceramide, autophagy flux, Lysosomal cell death, Immunology and Microbiology Section, Immune response, Immunity

## Abstract

Myeloid-derived suppressor cells (MDSCs) are immune suppressive cells that are hallmarks of human cancer. MDSCs inhibit cytotoxic T lymphocytes (CTLs) and NK cell functions to promote tumor immune escape and progression, and therefore are considered key targets in cancer immunotherapy. Recent studies determined a key role of the apoptosis pathways in tumor-induced MDSC homeostasis and it is known that ceramide plays a key role in regulation of mammalian cell apoptosis. In this study, we aimed to determine the efficacy and underlying molecular mechanism of ceramide in suppression of MDSCs. Treatment of tumor-bearing mice with LCL521, a lysosomotropic inhibitor of acid ceramidase, significantly decreased MDSC accumulation *in vivo*. Using a MDSC-like myeloid cell model, we determined that LCL521 targets lysosomes and increases total cellular C16 ceramide level. Although MDSC-like cells have functional apoptosis pathways, LCL521-induced MDSC death occurs in an apoptosis- and necroptosis-independent mechanism. LCL521 treatment resulted in an increase in the number of autophagic vesicles, heterolysosomes and swollen ERs. Finally, concomitant inhibition of cathepsin B and cathepsin D was required to significantly decrease LCL521-induced cell death. Our observations indicate that LCL521 targets lysosomes to activate cathepsin B and cathepsin D, resulting in interrupted autophagy and ER stress that culminates in MDSC death. Therefore, a ceramidase inhibitor is potentially an effective adjunct therapeutic agent for suppression of MDSCs to enhance the efficacy of CTL-based cancer immunotherapy.

## INTRODUCTION

Myeloid-derived suppressor cells (MDSCs) are potent immune suppressive cells that are induced by various pathological conditions, particularly by cancer [1-8]. In human cancer patients and mouse tumor models, massive accumulation of MDSCs is a hallmark of tumor progression [9-14]. It has been shown that MDSCs use diverse mechanisms to inhibit the effector function of T cells and NK cells [3, 15, 16]. Moreover, MDSCs modulate the tumor microenvironment rendering it favorable for angiogenesis, tumor growth, and progression through non-immunologic ways, which complement the pro-tumor activity of tumor-associated macrophages and neutrophils [15, 17]. Therefore, by virtue of their functions as suppressors of anti-tumor immunity and producers of growth enhancers, MDSCs are considered as key targets in cancer immunotherapy [7, 13, 18-23].

The mechanism underlying MDSC differentiation has been the subject of extensive studies, and it has been well-documented that MDSCs are originated in the bone marrow and are recruited to peripheral sites and the tumor microenvironment by inflammatory factors [11, 19, 24, 25]. Various pro-inflammatory factors, including IL-1β, IL-6, IL-10, prostaglandin E_2_ (PGE_2_), S100A8/9 proteins, GM-CSF, VEGF and HIF-1α, have been linked to MDSC accumulation [2, 26-30]. These studies clearly established the concept that tumor-induced inflammatory factors mediate MDSC accumulation in tumor-bearing hosts. Accordingly, current approaches to suppress MDSCs have been largely focused on inhibition of MDSC differentiation and proliferation [31, 32].

As in T cells, the homeostasis of MDSCs seems to be regulated, at least in part, by the Fas-mediated apoptosis pathway. It has been shown that host cytotoxic T lymphocytes (CTLs), once activated, use FasL to induce apoptosis of MDSCs to regulate their homeostasis [33]. It should be noted that MDSCs are generally sensitive to apoptosis induction, including FasL- and TRAIL-induced apoptosis [7, 34]. However, tumor-induced MDSCs exhibit altered levels of apoptosis regulatory proteins and decreased Fas receptor, and are therefore less sensitive to FasL-induced apoptosis than MDSC-like cells from tumor-free mice [35]. Therefore, therapeutic approaches to target MDSC death pathways are potentially an effective treatment strategy to suppress MDSCs in tumor-bearing hosts.

Sphingolipids are bioeffectors that mediate various cellular processes, including cell death [36-39]. Ceramide is the central molecule of sphingolipid metabolism pathways and has been shown to be effective in inducing tumor cell apoptosis. However, whether ceramide regulates MDSC apoptosis is unknown. In this study, we utilized LCL521 (prodrug of B13), a lysosomal inhibitor of acid ceramidase, and characterized its efficacy in suppressing MDSCs in tumor-bearing mice *in vivo* and in cultured cells *in vitro* and identified key effectors of the underlying molecular mechanism of suppression. We show that LCL521 induces MDSC death through a mechanism that is independent of apoptosis and necroptosis. Instead, LCL521 targets lysosomal cathepsins B and D to disrupt macroautophagy (hereafter referred to as autophagy) flux and induce endoplasmic reticulum (ER) stress, resulting in MDSC cell death.

## RESULTS

### LCL521 suppresses MDSC accumulation in tumor-bearing mice

**Figure 1:**
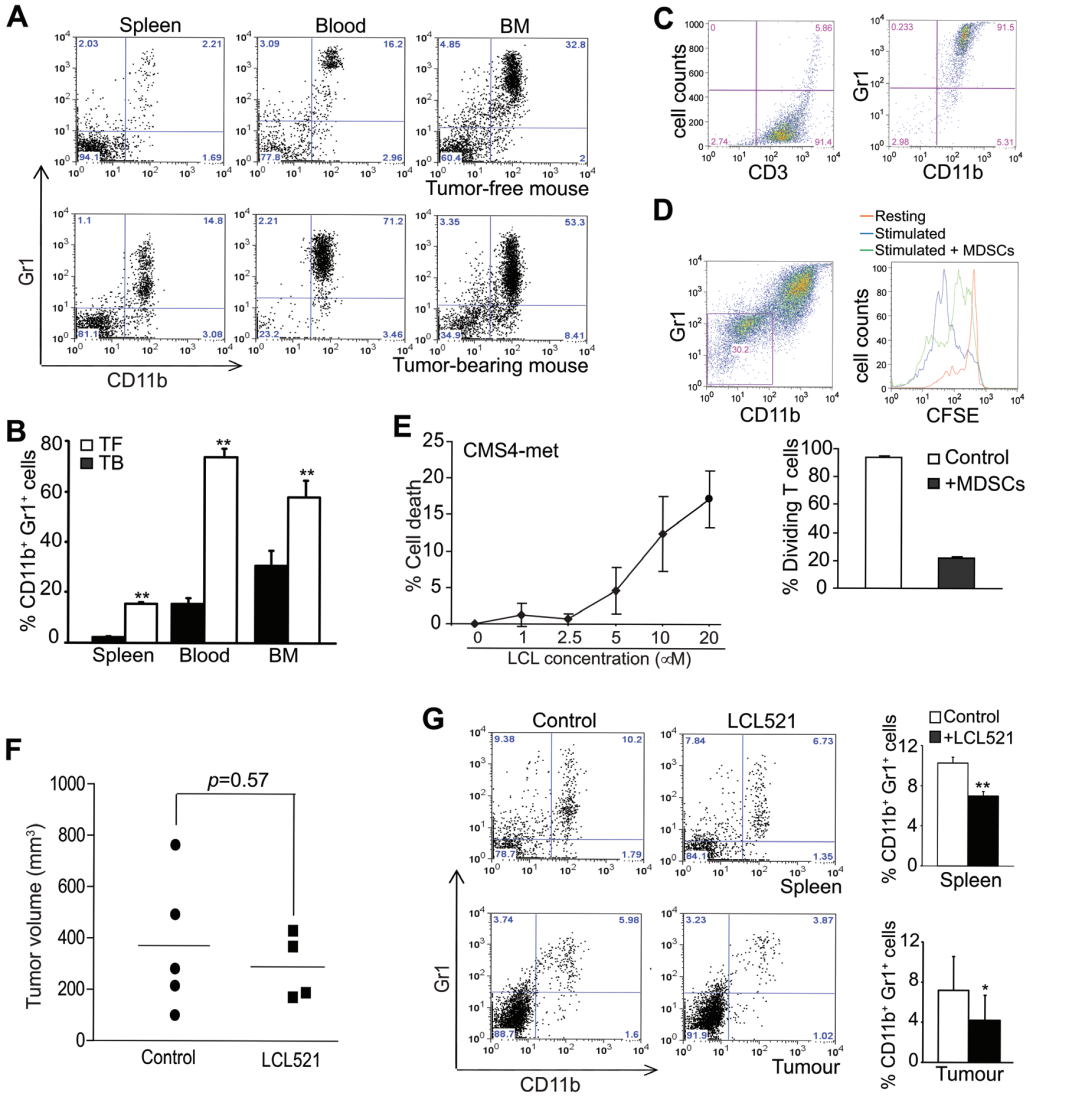
LCL521 suppresses MDSC accumulation in tumor-bearing mice in vivo **A.** CMS4-met tumor cells were injected into BALB/c mice sc. Spleen, blood and bone marrow (BM) were collected from tumor-free and tumor-bearing mice thirty days after tumor cell injection. Cells were stained with CD11b- and Gr1-specific mAbs and analyzed by flow cytometry. Shown are representative plots of MDSCs from one mouse of three mice in each group. **B.** The CD11b^+^Gr1^+^ MDSCs were quantified as percentage of total cells. Column: mean, Bar: SD. **C.** CD3^+^ T cells were purified from spleens of tumor-free BALB/c mice, and MDSCs were purified from spleens of tumor-bearing mice as described in the materials and methods. The purified cells were analyzed by flow cytometry for purity. The purities of the purified cells are indicated in the plots. **D.** The purified CD3^+^ T cells (1.5 x 10^5^ cells/well) were labelled with CFSE, and then cultured in anti-CD3 and anti-CD28-coated plates in the absence or presence of purified MDSCs (1x10^6^ cells/well) for 3 days. Cell culture mixtures were stained with anti-CD11b and anti-Gr1 mAbs. CD11b^-^Gr1^-^ cells were gated (left panel) and analyzed for CFSE intensity (right panel. The unstimulated (resting), and stimulated T cells in the absence (stimulated) or presence (stimulated + MDSCs) of MDSCs were overlayed for CFSE intensity. Shown is a representative images of CSFE intensity of the three groups of cells (right panel). Cells were quantified based on CFSE intensity and presented in the bottom panel. **E.** Mouse tumor cell line CMS4-met was cultured in the presence of LCL521 at the indicated doses for approximately 24 hours. Cells were stained with PI and analyzed for cell death. % cell death is calculated as % PI^+^ cells. **F.** CMS4-met tumor cells were injected into BALB/c mice. Thirty days later, tumor-bearing mice were treated with LCL521 at a dose of 75 mg/kg body weight every two days twice. Shown are tumor volumes in control and LCL521-treated mice. **G.** Spleens and tumors were collected from control and LCL521-treated tumor-bearing mice. Spleen cells were stained with CD11b- and Gr1-specific mAbs and analyzed by flow cytometry. Tumors were digested with collagenase solutions to make single cell suspension. The tumor tissue single cell suspension was then stained with CD11b- and Gr1-specific mAbs and analyzed by flow cytometry. Shown are representative results of one of three pairs of untreated and LCL521-treated tumor-bearing mice. The % CD11b^+^ Gr1^+^ MDSCs were quantified and presented at the right. Column: Mean, Bar: SD.

To determine whether inhibition of acid ceramidase increases MDSC death, we made use of a tumor-bearing mouse model and a lysosomotropic inhibitor of acid ceramidase [40, 41]. CMS4-met tumor cells were injected into BALB/c mice s.c. to establish tumor. Analysis of the tumor-bearing mice indicated, as expected, that CD11b^+^Gr1^+^ MDSCs massively accumulated in spleen, blood and BM (Figure 1A & 1B). To functionally validate these tumor-induced MDSCs are immune suppressive, MDSCs and T cells were co-cultured and analyzed for T cell proliferation. CD3^+^ T cells were purified from spleens of BALB/c mice and MDSCs were purified from tumor-bearing BALB/c mice. The purities of purified CD3^+^ T cells and CD11b^+^Gr1^+^ MDSCs are greater than 90% (Figure 1C). CD3^+^ T cells were labelled with CFSE and cultured in anti-CD3 and anti-CD28-coated plates. MDSCs were added to the T cell culture and T cell proliferation was analyzed 3 days later. The purified primary MDSCs apparently survived the 3 day culture (Figure 1D). Analysis of T cell CFSE intensity indicates that, as expected, T cell divided under the anti-CD3 and anti-CD28 stimulation conditions (Figure 1D). MDSCs dramatically inhibited T cell proliferation (Figure 1D).

To determine whether LCL521 suppresses MDSC accumulation *in vivo*, we treated tumor-bearing mice with LCL521 and analyzed MDSC accumulation. LCL521 has been shown to exhibit direct tumor suppression activity [42]. Indeed, LCL521 effectively induces mouse tumor cell death *in vitro* (Figure 1E). Because tumor sizes determine MDSC accumulation level [43] and LCL521 may suppress tumor growth to indirectly suppress MDSC accumulation, we injected tumor cells to mice and let tumor grow to close to maximal size allowed by the animal protocol. The rationale is that LCL521 treatment will not significantly suppress tumor growth if the tumor burden is extensive. Therefore, the effect of LCL521 on MDSCs in tumor-bearing mice can be directly evaluated. The tumor-bearing mice were treated with LCL521 and tumor sizes were measured. LCL521 did not significantly decrease tumor size under this extensive tumor burden condition (Figure 1F). Analysis of MDSCs in spleens, blood and BM of the tumor-bearing mice revealed that MDSC levels were significantly lower in LCL521-treated mice as compared to control mice (Figure 1G). Therefore, we conclude that LCL521 can effectively suppress MDSC accumulation in tumor bearing host *in vivo*.

### LCL521 is a potent inducer of MDSC-like myeloid cell death

**Figure 2:**
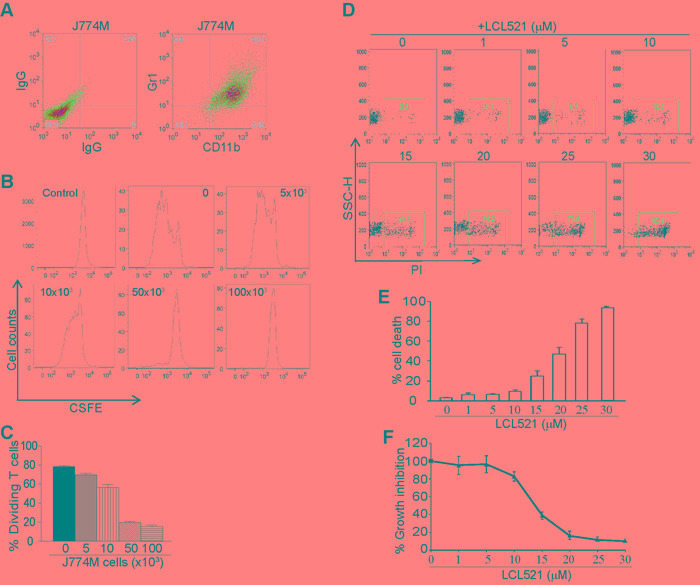
LCL521 exhibits potent cytotoxicity against MDSC-like cells in vitro **A.** J774M cells were stained with IgGs or CD11b- and Gr1-specific mAbs, and analyzed by flow cytometry. Shown are representative images of J774M phenotype. **B.** CD3^+^ T cells were purified from spleens of BALB/c mice and labeled with CFSE. The labelled T cells were then cultured in anti-CD3 and anti-CD28-coated plates in the presence of J774M cells at the indicated cell densities for 3 days. Cell mixtures were stained with CD11b-specific mAb and CD11b^-^ cells were gated for analysis for CFSE intensity by flow cytometry. Shown are representative images of CFSE intensity of T cells. Control indicates CFSE intensity of CFSE-labelled CD3^+^ T cells without stimulation. **C.** The dividing T cells as shown in B were calculated based on CFSE intensity. **D.** J774M cells were cultured in the presence of LCL521 at the indicated concentrations for approximately 24h. Cells were collected, stained with PI and analyzed by flow cytometry. Shown are representative results of one of three experiments. **E.** The % PI^+^ cells as shown in B were quantified. Column: mean; Bar: SD. **F.** J774M cells were cultured in the presence of LCL521 at the indicated concentrations for approximately 24h and analyzed by MTT assay. The viability of untreated cells was set at 100%. The viability of the treated cells was expressed as percentage over the control cells.

To elucidate the mechanism underlying LCL521-mediated suppression of MDSCs, we then used an *in vitro* MDSC-like cell model. J774 cells are monocyte macrophages of BALB/c mouse origin that express high levels of CD11b and low levels of Gr1. We sorted CD11b^+^Gr1^+^ cells from the parent J774 cells and established a CD11b^+^Gr1^+^ cell line, termed J774M (Figure 2A). To determine whether J774M cells functionally mimic MDSCs, we performed T cell-J774M cell co-culture. Purified CD3^+^ T cells were labelled with CFSE and cultured under stimulation conditions in the presence of various doses of J774M cells. J774M cells inhibited T cell proliferation (Figure 2B) and the inhibitory effect is J774M cell dose-dependent and a 0.33:1 ratio of J774M and T cells almost completely inhibited T cell proliferation *in vitro* (Figure 2C). Therefore, J774M cells phenotypically and functionally mimics tumor-induced MDSCs.

J774M cells were then used as an *in vitro* MDSC model and cultured in the presence of various concentrations of LCL521 for 24h, stained with PI, and analyzed by flow cytometry. LCL521 induced J774M cell death in a dose-dependent manner (Figure 2D & 2E). Next, a complementary approach was performed utilizing the MTT assay to identify effects of LCL521 on J774M cell viability. As expected, the MTT assay showed that a 24 hour treatment with LCL521 significantly decreased J774M cell viability in a dose-dependent manner (Figure 2F). Taken together, we conclude that LCL521 suppresses MDSC accumulation through inducing MDSC cell death.

### LCL521 targets lysosome to increase total cellular C16- ceramide level

**Figure 3:**
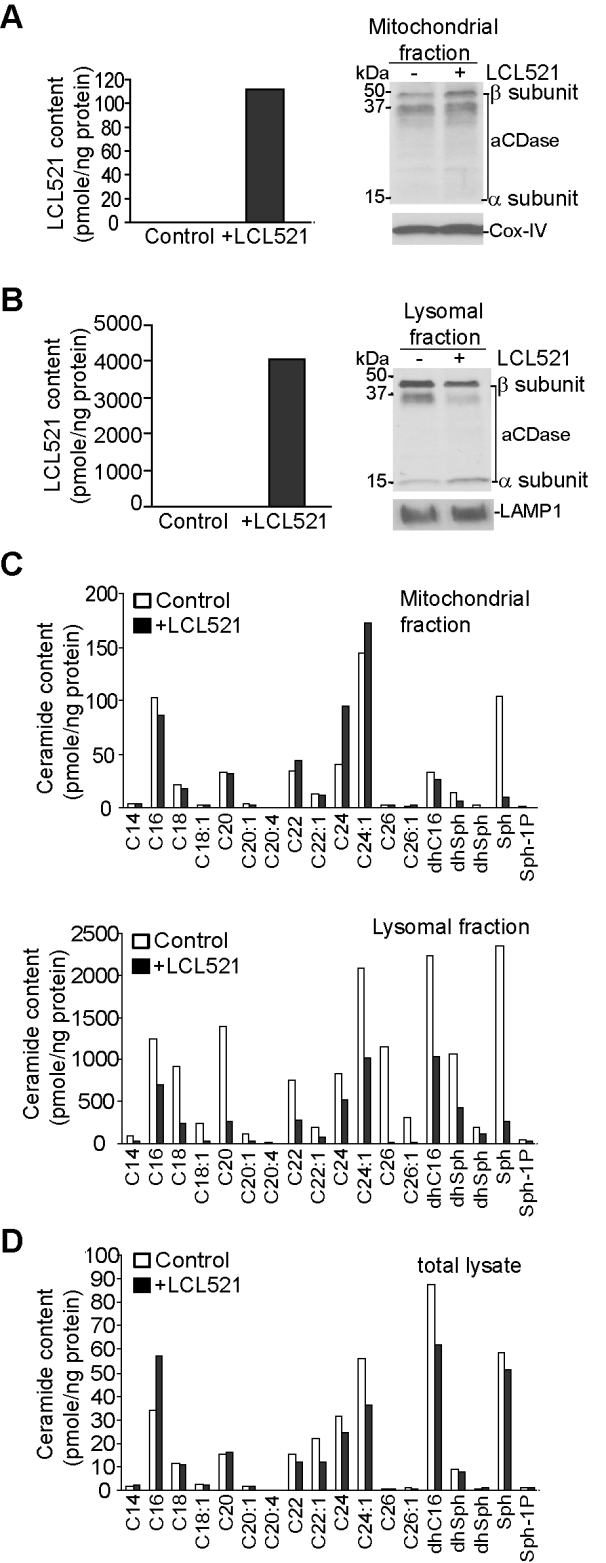
LCL521 targets lysosome and increase total cellular C16 ceramide **A.** J774M cells were cultured in the absence or presence of LCL521 (15 μM) for 30 min. Mitochondria were isolated using the cell fraction kit. Mitochondria were lysed in total lysis buffer and measured for LCL521 content. The mitochondria lysate was also analyzed by Western blotting for acid ceramidase protein levels. LAMP1 was used as normalization control. **B.** J774M cells were cultured in the absence or presence of LCL521 (15 μM) for 30 min. Lysosomes were isolated using Lysosome Enrichment Kit. Lysosomes were lysed in total lysis buffer and analyzed for ceramide content. The lysosome lysate was also analyzed by Western blotting analysis for acid ceramidase protein level (right panel). Cox-IV was used as normalization control. **C.** The mitochondria (top panel) and lysosome (bottom panel) lysates as shown in A and B, respectively, were also measured for ceramide contents. **D.** J774M cells were cultured in the absence or presence of LCL521 (5 μM) for 24h. Cells were then lysated to make total cellular lysates and analyzed for total cellular ceramide contents (bottom panel). The ceramide content was normalized to the total cellular protein level.

To identify the mechanism of LCL521-induced cell death, we next sought to determine the subcellular localization of LCL521. J774M cells were treated with LCL521. Mitochondria and lysosomes were isolated from control and treated cells. LCL521 levels were measured in the control and treated cells. LCL521 level was dramatically higher in the lysosomes compared to the mitochondria (Figure 3A & 3B). The levels of α and β subunits of acid ceramidase were not significantly different between control and LCL521-treated cells in mitochondria (Figure 3A). The β subunit of acid ceramidase protein increased after LCL521 treatment in lysosomes after LCL521 treatment (Figure 3B). Levels of most of the ceramide species were relatively low in mitochondria. LCL521 decreased C16 and sphingosine and increased C24 and C24:1 ceramide (Figure 3C). In stark contrast, levels of ceramides were much higher in the lysosomes than in mitochondria, and surprisingly, LCL521 treatment decreased almost all ceramides in the lysosomes (Figure 3C). Analysis of total cellular ceramide levels revealed that LCL521 increased total cellular C16 ceramide and decreased C22, C24, C24:1, dhC16 and sphingosine (Figure 3C).

These observations indicate that the lysosome is a major target of LCL521 action, and potentially identify C16 ceramide as one of the effector of LCL521 cytotoxic action [44].

### LCL521 induces MDSC death by an apoptosis and necroptosis-independent mechanism

**Figure 4:**
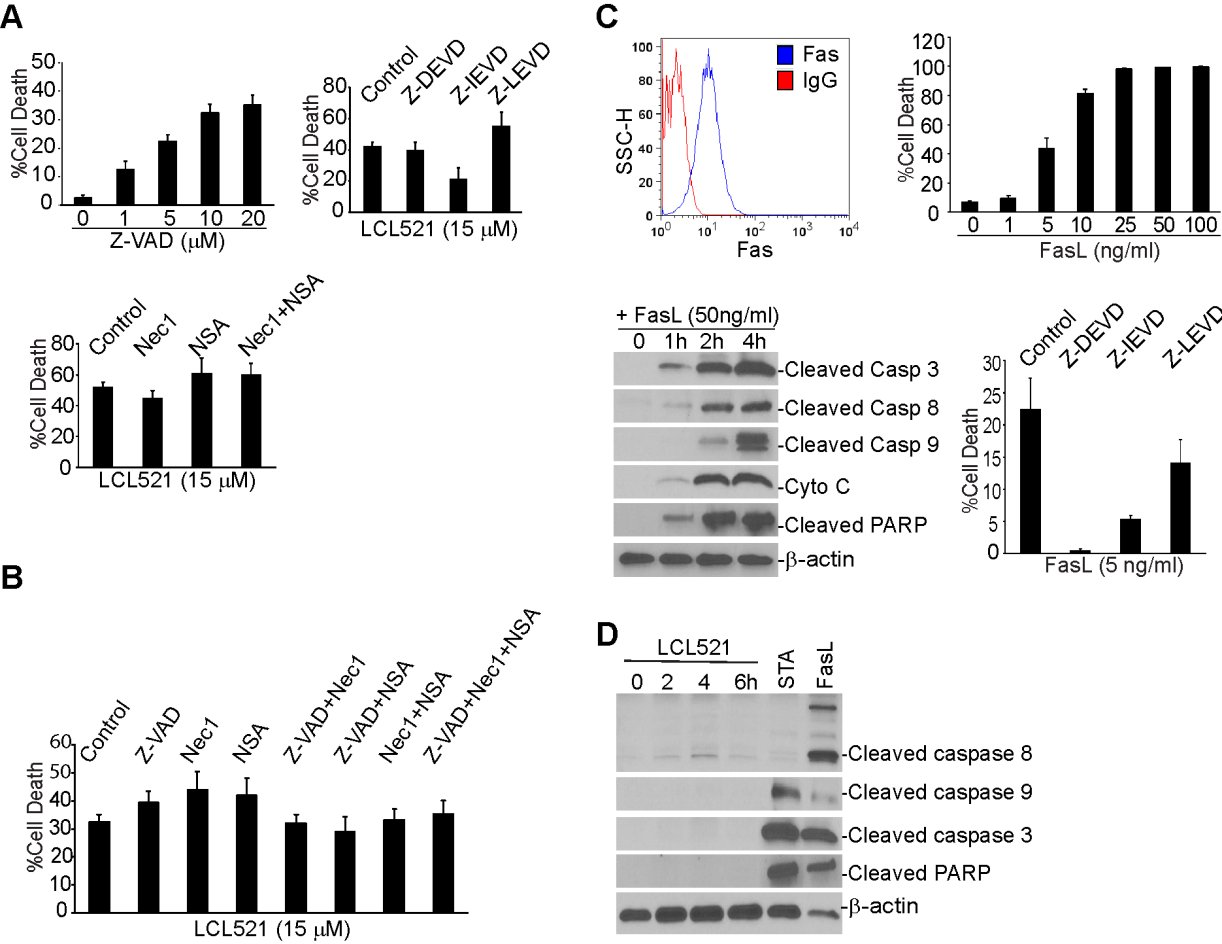
Ceramide mediates MDSC cell death through an apoptosis- and necroptosis-independent mechanism **A.** J774M cells were cultured in the presence of Z-VAD at the indicated dose (top left panel), LCL521 (5 μM) plus caspase (top right panel) or necroptosis (bottom panel) inhibitors as indicated, for approximately 24h. Cells were collected, stained with PI solution and analyzed by flow cytometry. % cell death is expressed as % PI^+^ cells. **B.** J774M cells were cultured in the presence of caspase and necroptosis inhibitors, either alone or in combinations as indicated, for 24h, stained with PI and analyzed by flow cytometry. % cell death is expressed as PI^+^ cells. **C.** J774M cells were stained with IgG isotype control or Fas-specific mAbs and analyzed by flow cytometry (top left panel). J774M cells were cultured in the presence of FasL at the indicated concentrations for approximately 24h. Cells were stained with PI and analyzed by flow cytometry for cell death (top right panel). Bottom left panel: J774M cells were treated with FasL for the indicated time and analyzed by Western blotting analysis for cleavage/activation of the indicated caspases, cytochrome C (Cyto C) release and PARP cleavage. Bottom right panel: J774M cells were cultured in the presence of the indicated caspase inhibitors for approximately 24h. Cells were then stained with PI and analyzed by flow cytometry. **D.** J774M cells were treated with LCL521 (5 μM) for the indicated time. J774M cells were also treated with STA and FasL for 6h. Cytosol fractions were then prepared from the cells and analyzed by Western blotting analysis for the indicated proteins. Western blots in C and D were cropped to improve the clarity and conciseness of the results. Gels were run and blotted under the same experimental conditions.

Having determined that LCL521 induces MDSC cell death, we next sought to characterize the mechanism of cell death with a focus on apoptosis and necroptosis as the potential death pathways. To determine if LCL521 induces apoptosis, J774M cells were cultured in the presence of various concentrations of the pan-caspase inhibitor Z-VAD. Interestingly, Z-VAD treatment induced J774M cell death in a dose-dependent manner (Figure 4A). We therefore used inhibitors of individual caspases, including caspase 3, caspase 8 and caspase 9. J774M cells were incubated in the presence of LCL521 plus or minus these caspase inhibitors and cell death was quantitated. LCL521 induced J774M cell death was not ameliorated by the presence of caspase 3, 8 or 9 inhibitors (Figure 4A). Next, we sought to determine whether LCL521 induces J774M cell death through necroptosis, J774M cells were treated with LCL521 plus Nec1, NSA, or Nec1 and NSA together. Cell death was then quantitated. As expected, LCL521 induced widespread cell death, but neither the RIP1 inhibitor Nec1 nor the RIP3 inhibitor NSA had any effect on LCL521-induced J774M cell death (Figure 4A).

It is generally believed that inhibition of apoptosis promotes necroptosis [45]. However, we observed here that inhibition of either apoptosis or necroptosis did not affect LCL521-induced J774M cell death. To determine whether apoptosis and necroptosis compensate each other in LCL521-induced J774M cell death, we treated J774M cells with LCL521 and inhibitors of both apoptosis and necroptosis. If apoptosis and necroptosis compensate for each other in LCL521-induced cell death, then blocking both apoptosis and necroptosis should diminish LCL521-induced cell death. Analysis of J774M cells indicated that inhibition of apoptosis with Z-VAD and inhibition of necroptosis with Nec1 and NSA does not reverse LCL521-induced J774M death (Figure 4B). Therefore, we conclude that LCL521 does not induce apoptosis and necroptosis in J774M cells.

To determine whether the apoptosis signaling pathway is functional in J774M cells, we analyzed J774M cell sensitivity to FasL. Flow cytometry analysis revealed that the death receptor Fas is expressed on the J774M cell surface and J774M cells are sensitive to FasL-induced apoptosis (Figure 4C). As expected, FasL induced rapid activation of caspases 3, 8 and 9, cytochrome C release and apoptosis as evidenced by cleaved PARP (Figure 4C). Furthermore, inhibitors for caspases 3, 8 and 9 all inhibited FasL-induced J774M cell death (Figure 4C). To determine whether LCL521 activates caspases, we treated J774M cells with LCL521 and analyzed caspase activation using FasL and staurosporine as positive controls. Staurosporine and FasL both induced rapid caspase activation in J774M cells, but LCL521 did not induce detectable cleavage of caspase 3, 8 or 9 or PARP (Figure 4D).

**Figure 5:**
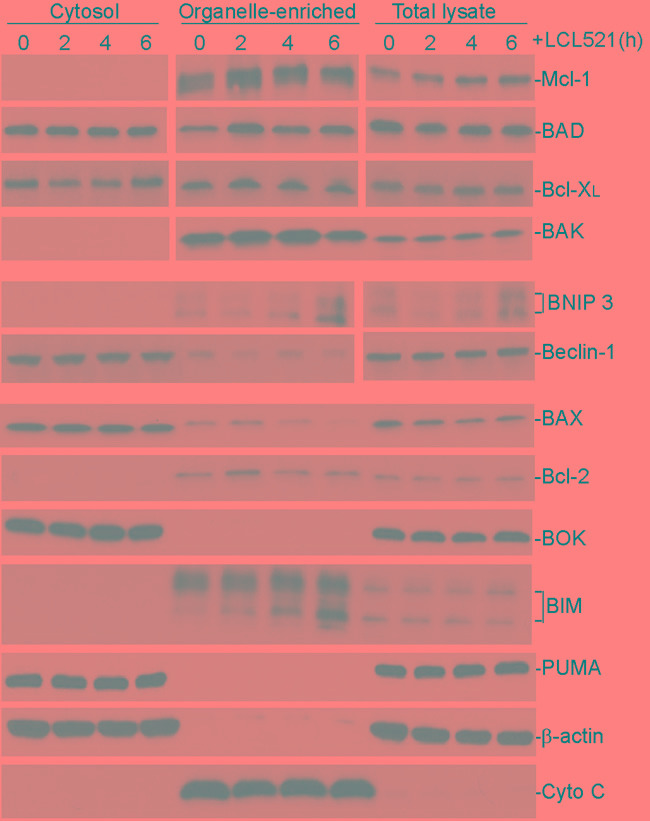
Ceramide does not alter apoptosis regulatory protein level in MDSC-like myeloid cells Cells were treated with LCL521 (15 μM) and harvested at the indicated time points. Cytosol, mitochondrial fractions and total lysate were prepared and resolved in 4-20% SDS-polyacrylamide gels, and then analyzed by Western blotting analysis using antibodies for the indicated proteins. β-actin is used as normalization control for the cytosol fractions and total lysate. Cyto C is used as normalization control for the mitochondrial fractions. Western blots were cropped to improve the conciseness of the results. Gels were run and blotted under the same experimental conditions. Blots from different gels are separated by white space.

It is well-documented that ceramide mediates expression of apoptosis regulatory genes [44, 46-48]. We then treated J774M cells with LCL521 and prepared cytosol and mitochondria-enriched fractions. Western blotting analysis of the commonly known apoptosis regulators indicated that LCL521 did not alter these apoptosis regulators in J774M cells (Figure 5). These observations thus strength our findings that LCL521 induces MDSC death through an apoptosis- and necroptosis-independent mechanism.

### Inhibition of acid ceramidase increases autophagosome accumulation and increases ER stress

**Figure 6:**
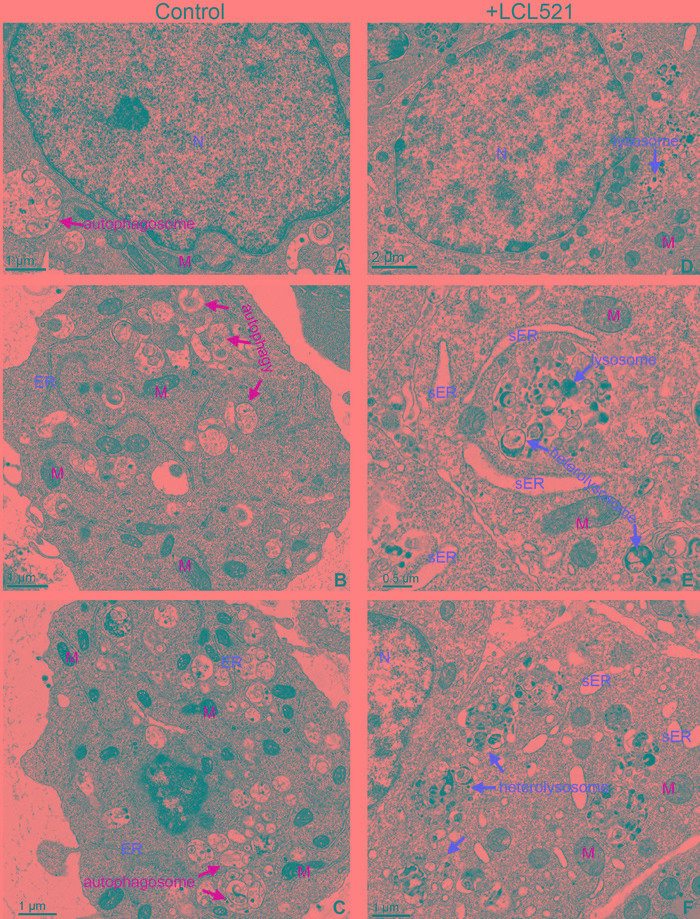
LCL521 treatment disrupts autophagy to activate lysosomal degradation of stressed ER J774M cells were either untreated **A.**-**C.** or treated with LCL521 (5 μM) **D.**-**F.** for approximately 24h. Cells were then fixed and embedded in resin. Thin sections were stained with uranyl acetate and lead citrate. Ultracellular structures were observed in a transmission electron microscope. M: mitochondrion, N: nucleus, sER: swollen ER,

To determine which cell death mechanism was induced by LCL521 in J774M cells, we then analyzed cellular ultrastructure in J774M cells by electron miscroscopy following LCL521 treatment. Nuclei were mostly normal in growing J774M cells and very few cells exhibited condensed chromatin (Figure 6A). The cytoplasm of untreated cells contained abundant autophagosomes, often inside multivesicular bodies typical of secondary lysosomes (Figure 6B & 6C). The ER appeared mostly normal (Figure 6B & 6C) with only a few cells showing a small amount of swollen ER. Many lysosomes were visible inside multivesicular bodies and appeared to be involved in the breakdown of autophagosomes (Figure 6B & 6C). Mitochondria exhibited normal morphology (Figure 6B & 6C). In LCL521-treated J774M cells nuclear morphology was mostly normal with few signs of condensed chromatin, suggesting that LCL521 does not induce apoptotic cell death (Figure 6D). However, in the cytoplasm of LCL521-treated J7784M cells, grossly elevated numbers of autophagic vesicles were observed compared to untreated cells. Many of these autophagic vesicles were located in association with lysosomes (Figure 6E & 6F). Normal ER morphology was rare with widespread swollen ER visible in almost all cells (Figure 6E & 6F), indicating ubiquitous ER stress. Greatly elevated numbers of heterolysosomes were also observed in LCL521-treated cells. These heterolysosomes were often located in close proximity to swollen ER (Figure 6E & 6F), indicating possible lysosomal degradation of swollen ER. Most mitochondria in LCL521-treated cells appeared normal, but some enlarged mitochondria with swollen christae were visible and were often associated with swollen ER (Figure 6E). Taken together, these observations indicate that autophagy is active in J774M cells under normal conditions while LCL521 targets lysosomes to alter structures of autophagosomes, lysosome and ER.

### LCL521 induces MDSC death through interrupting autophagy flux

**Figure 7:**
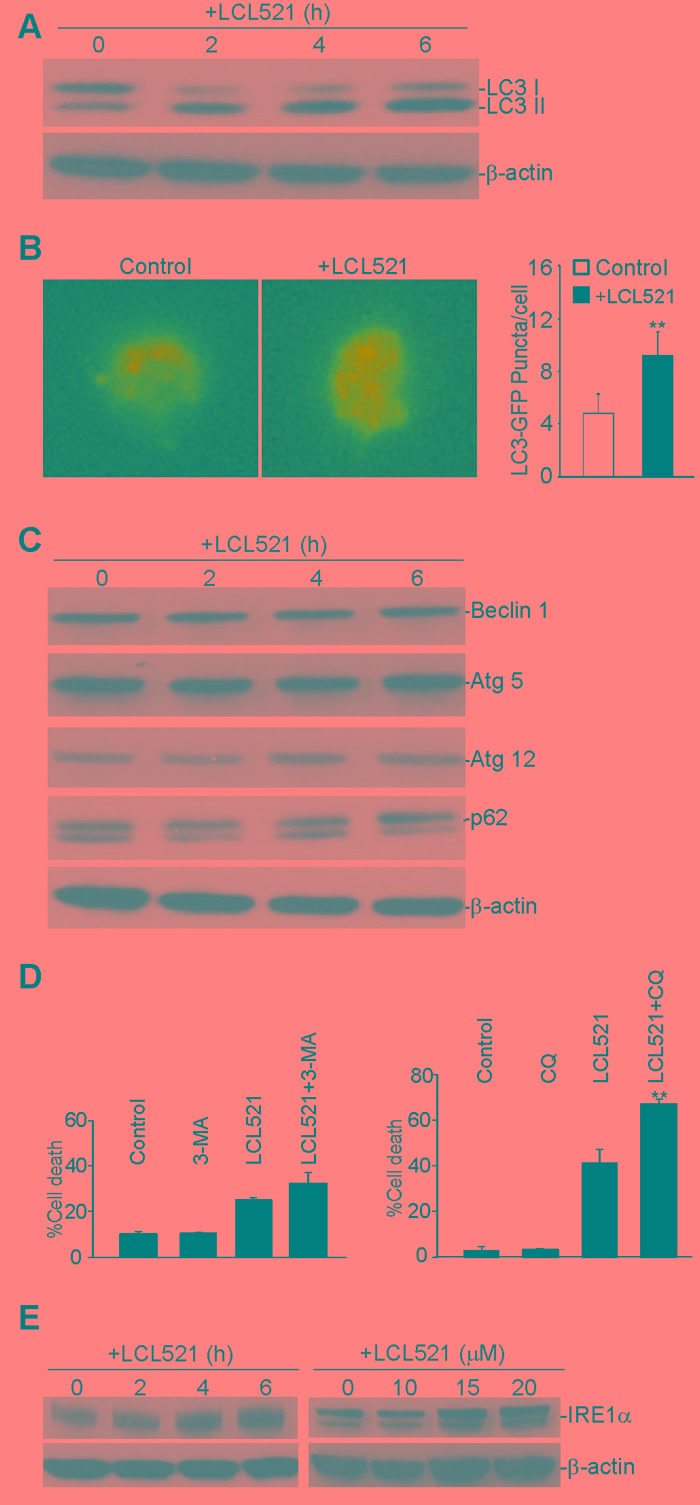
Inhibition of acid ceramidase increase autophagy in MDSC-like myeloid cells **A.** J774M cells were treated with LCL521 (15 μM) for the indicated time and analyzed by Western blotting for LC3 protein level. β-actin was used as normalization control. **B.** J774 cells were transfected with GFP-LC3-expressing vector for 24h, followed by treatment with LCL521 (5 μM) for 24h. Shown are representative images of GFP fluorescence (left panel). The number of GFP puncta were counted and presented at the right. Column: mean; Bar:SD. **C.** J774M cells were cultured in the presence of LCL521 (15 μM) and analyzed for the indicated proteins by Western blot at the indicated time points. β-actin was used a normalization control. **D.** J774M cells were cultured in the presence of LCL521 (15 μM) plus 3-MA and CQ either alone or in combination, as indicated, for 24 h and analyzed for cell death by PI staining. Column: mean; Bar: SD. **E.** J774M cells were cultured in the presence of LCL521 (15 μM) for the indicated time or cultured in the presence of various concentrations of LCL521 as indicated, and analyzed for IRF1a protein level by Western blotting. β-actin was used as normalization control. Western blots in C and E were cropped to improve the conciseness of the results. Gels were run and blotted under the same experimental conditions.

Ceramide mediates autophagy [49]. Our above cellular ultrastructure analysis indicated that LCL521 caused grossly elevated numbers of autophagic vesicles in the cytoplasm of J774M cells, suggesting interruption of autophagic flux in J774M cells. To examine the induction of autophagy after LCL521 treatment, we assessed the level of microtubule-associated protein 1 light chain 3 (LC3) in J774M cells by Western blotting analysis. Conversion of LC3 I to the lipidated form (LC3-II) by addition of phosphatidylethanolamine is essential for the formation of autophagosomes and is considered as a reliable biochemical marker of autophagosome formation and accumulation. Levels of LC3 II increased rapidly after LCL521 treatment (Figure 7A), indicating accumulation of autophagosomes soon after LCL521 treatment. To validate this finding, we then ectopically expressed LC3-GFP in J774M cells and analyzed GFP puncta. In contrast to the cytoplasmic localization of LC3 I, LC3 II associates with both the outer and inner membranes of the autophagosome. After fusion with the lysosome, LC3 on the outer membrane is cleaved off by Atg4, and LC3 on the inner membrane is degraded by lysosomal enzymes, resulting in very low LC3 levels in the autolysosome. Thus, endogenous GFP-LC3 should be present either as a diffuse cytoplasmic pool external to autophasomes or as puncta that represent autophagosomes. Therefore, the number of punctate GFP structures per cell is an accurate measure of autophagosome number [50]. J774M cells exhibited some GFP punctate structures, indicating rapid fusion of autophagosmes and lysosomes. However, LCL521 treatment significantly increased the number of GFP puncta (Figure 7B), suggesting interrupted autophagosome degradation by lysosomes.

To elucidate the mechanism underlying LCL521 induction of autophagy interruption in J774M cells, we examined biochemical markers that are involved in regulation and formation of autophagosomes. It is known that Beclin 1 plays a key role in initiation of autophagy [51], and conjugation of autophagy proteins 5 and 12 (Atg5-Atg12) is essential for autophagosome elongation[52]. However, LCL521 treatment neither altered beclin 1 protein level nor changed Atg5 and Atg12 protein levels (Figure 7C). These observations indicated that LCL521 did not affect autophagosome formation in J774M cells.

Increased LC3 II could be due to either increased autophagosome formation or decreased autophagosome degradation. P62 is an adapter protein that mediates ubiquitinated cargo entry to the autophagosome. P62 is degraded alongside its cargos by auotphagosomes, and accumulation of p62 is thus an indicator of impaired autophagic degradation [53]. However, treatment of J774M cells did not increase p62 level (Figure 7C). Therefore, LCL521 did not alter autophagosome degradation in J774M cells (Figure 7C).

Autophagy can either promote cell survival or death depending on the cellular context [54]. We then analyzed LCL521-induced J774M cell death in the presence of autophagy inhibitors 3-Methyladenine (3-MA) and chloroquine (CQ). 3-MA inhibits autophagy by blocking autophagosome formation through the inhibition of class III phosphatidylinositol 3-kinases (PI3K) [55] and thus is an inhibitor autophagy in the early stages. CQ targets lysosomes to cause an increased lysosomal pH resulting in inactivation of lysosomal hydrolysates to increase autolysosomes accumulation. CQ is thus is a late autophagy inhibitor [56]. Treatment of J774M cells with 3-MA did not induce J774M cell death, indicating that although autophagy is active in J774M cells (Figure 6), autophagy is not essential for J774M cell survival or there is a compensatory cell survival pathway. 3-MA slightly increased J774M cell sensitivity to LCL521-induced cell death. Treatment of J774M cells with CQ induced a small degree of J774M cells death and significantly increased J774M cells to LCL521-induced cell death (Figure 7D). These observations indicate that autophagy promotes J774M survival and LCL521 may interrupt autophagy flux at the late stage to induce J774M cell death.

### LCL521 induces ER stress

In addition to altered autophagy flux, our cellular ultrastructural analysis revealed that LCL521 induced widespread swollen ER. Although autophagy is primarily a lysosome-mediated degradation pathway for recycling and elimination of proteins and damaged organelles, it has emerged that autophagy also protects against ER stress and these two processes are dynamically interconnected [57-61]. To determine whether LCL521-induced swollen ER (Figure 6E & 6F) was associated with ER stress, we analyzed IRE1a, an ER stress sensor, by Western blotting. LCL521 treatment rapidly increased IRE1a levels in a dose-dependent manner in J774M cells (Figure 7E).

### LCL521 induces MDSC cell death through a cathepsin-dependent mechanism

**Figure 8:**
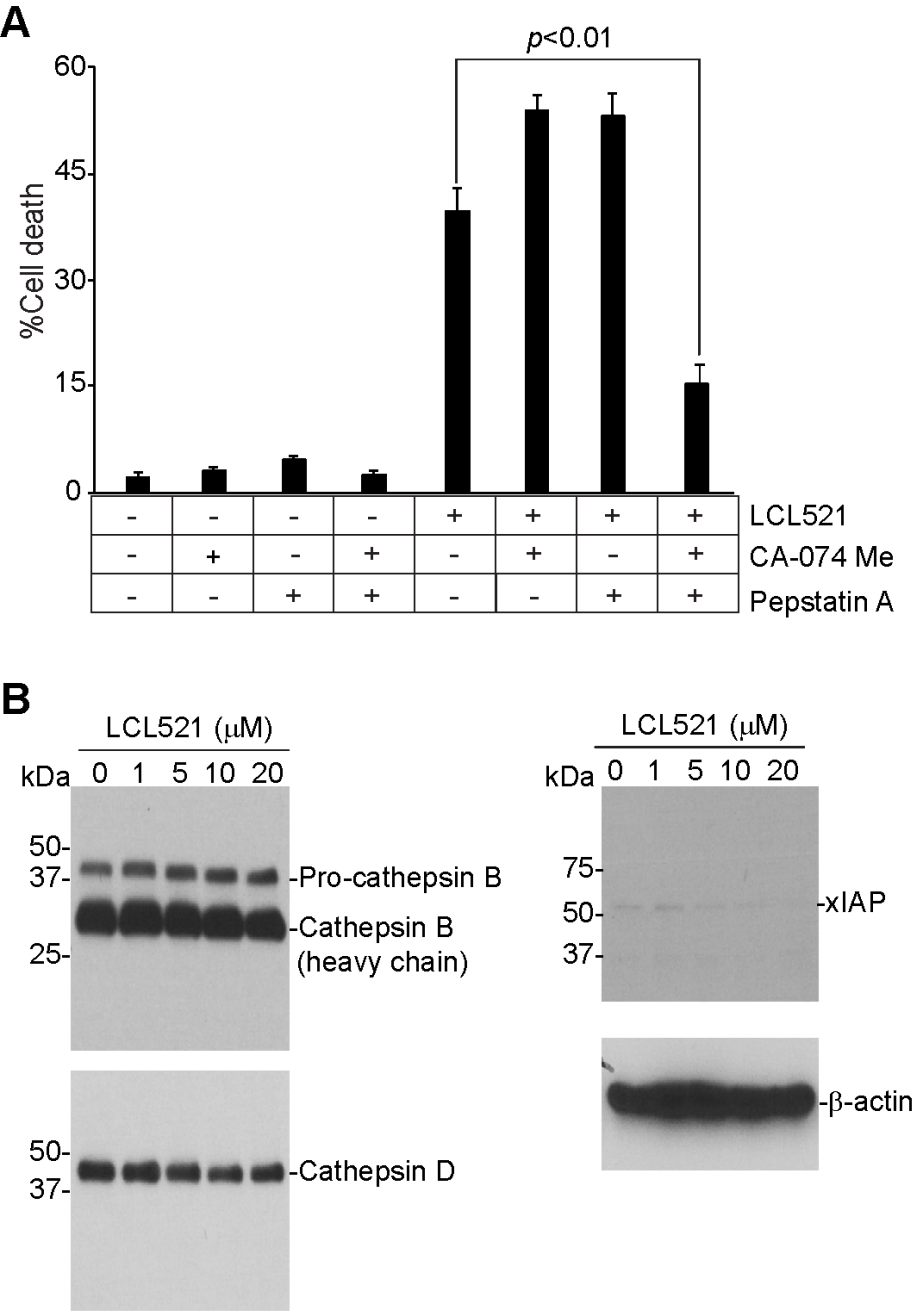
LCL521 induces MDSC-like myeloid cell death through a cathepsin-dependent mechanism **A.** J774M cells were pre-treated with CA-074 Me (4 μM) or Pepstatin A (4 μM), respectively for 30 min, and then either untreated or treated with LCL521 (15 μM) for approximately 24h. Cells were stained with PI and analyzed by flow cytometry. Cell death is expressed as % PI^+^ cells. Column: mean; Bar: SD. **B.** J774M cells were cultured in the presence of LCL521 at the indicated doses for approximately 24 hours. Cell total lysates were analyzed by Western blotting using antibodies that are specific for the indicated proteins.

LCL521 targets lysosomes (Figure 3B) and since cathepsins are lysosomal proteases that are known to mediate cell death [62, 63], we hypothesized that LCL521 might target lysosomal cathepsins to induce J774M cell death. To test this hypothesis, J774M cells were treated with LCL521 in the presence of inhibitors for cathepsin B (CA-074me) and cathepsin D (Pepstatin A), respectively. Inhibition of either cathepsin B or cathepsin D alone did not block LCL521-induced cell death. However, simultaneous inhibition of both cathepsin B and cathepsin D significantly decreased LCL521-induced cell death (Figure 8A). Western blotting analysis indicated that LCL521 does not alter the levels of cathepsin B and cathepsin D proteins (Figure 8B), suggesting that LCL521 activates the enzymatic activity but not the protein levels of cathepsin B and cathepsin D. Previous studies have shown that ceramide induces xIAP degradation to mediate cell death [44, 46, 48]. Western blotting analysis indicates that although LCL521 treatment decreased xIAP protein level in J774M cells, xIAP protein level is essentially low in J774M cells (Figure 8B), suggesting that xIAP may not play a significant role in MDSCs. Taken together, our observations indicate that LCL521 induces J774M cell death through a cathepsin B- and cathepsin D-dependent mechanism and that cathepsin B and cathepsin D compensate each other in LCL521-induced cell death.

## DISCUSSION

Ceramides are known to function as general cell death or survival regulators in various types of cells [36-39]. One of the mechanisms underlying ceramide regulation of cell death is enhancement of death receptor clustering to increase extrinsic apoptosis [64]. Ceramide also regulates the expression of apoptosis regulatory genes, including Bax, Bak, xIAP, Bcl-x, to increase the sensitivity of various types of mammalian cells to apoptosis. LCL521 is an acid ceramidase inhibitor that inhibits acid ceramidase to increase ceramide accumulation and sensitizes tumor cells to apoptosis [44, 46-48]. B13, the parent form of LCL521, has also been shown to increase ceramide levels in tumor cells to induce tumor cell apoptosis [41]. However, in this study, we observed that although LCL521 induces MDSC-like J774M cell death *in vitro* and suppresses MDSC accumulation in tumor-bearing mice *in vivo*, LCL521-induced cell death is apoptosis-independent. Consistent with the apoptosis-independent mechanism, LCL521 did not alter the expression levels of the known apoptosis regulatory proteins. Therefore, our data demonstrated that LCL521 induces MDSC death through a mechanism that is different to that in tumor cells.

Under normal culture conditions, the cytoplasm of J774M cells contains high levels of autophagosomes. The LC3I/II ratio and level of LC3-GFP puncta are low. In addition, lysosomes and ERs appear all normal. These observations indicate that active autophagy occurs in MDSCs to maintain normal cellular homeostasis. Analysis of cellular ultrastructure revealed that LCL521 primarily targets three cellular organelles: autophagosomes, lysosomes and ER. The increased LC3II/I ratio and LC3-GFP puncta indicate that LCL521 either stimulates autophagosome initiation or inhibits autophagosome degradation. Our observations that LCL521 did not change Beclin 1 and Atg5-Atg12 levels suggest that LCL521 does not alter autophagosome initiation in J774M cells. However, neither did LCL521 alter p62 protein level in J774M cells, suggesting that LCL521 does not impair autophagosome degradation. After LCL521 treatment, normal autophagosomes numbers are reduced while autophagic vesicle numbers are increased, suggesting interruption of normal autophagosome degradation leading to generation of abnormal autophagic vesicles. Although many of the LCL521-induced autophagic vesicles were located in association with lysosomes, lysosomes apparently failed to degrade these autophagic vesicles. Therefore, instead of inducing autophagosome accumulation or inhibiting autophagosome degradation, LCL521 induces accumulation of autophagic vesicles which may represent dysfunctional autophagosomes. This type of dysfunctional autophagosome/autophagic vesicle may impair normal autophagic flux to induce cell death and thus represent a novel cell death mechanism in MDSCs.

LCL521 is a lysosomotropic inhibitor of acid ceramidase [40, 41, 65]. As an acid ceramidase, LCL521 inhibits acid ceramidase to cause ceramide accumulation to enhance tumor cell sensitivity to apoptosis [41]. However, treatment of LCL521 resulted in decreased ceramide level in lysosomes. Because LCL521 treatment leads to altered lysosome structure, it is possible that LCL521 induces lysosome structure changes to cause ceramide leakage from lysosome. This notion is supported by the observation that total cellular C16 ceramide, a ceramide known to promote cell death [66], is increased in LCL521-treated J774M cells. It has been shown that ceramide binds to cathepsin to activate their enzymatic activity [46, 67]. In natural killer/T lymphoma cells, ceramide can activate cathepsin B to degrade xIAP to induce caspase-dependent apoptosis [46]. However, in MDSCs, xIAP protein level is low. Although LCL521 did decrease xIAP protein level, it is unlikely that xIAP plays a significant role in MDSC survival. Furthermore, LCL521-induced MDSC cell death does not require caspase activation. Instead, we observed that inhibition of cathepsin B and cathepsin D diminished LCL521-induced MDSC cell death. Therefore, it is reasonable to assume that LCL521 inhibits acid ceramidase to increase ceramide accumulation to activate cathepsins B and D to induce MDSC cell death. LCL521 is a prodrug of B13 and B13 is structurally similar to ceramide [40, 41]. Therefore, it is also possible that LCL521 itself may actually function as a ceramide analog in the lysosome to bind to cathepsins B and D to activate these two proteases. The downstream targets of cathepsin B and cathepsin D in MDSCs remain to be identified.

It is also possible that LCL521 induces lysosomal cell death of MDSCs. Lysosomal membrane permeabilization and the resultant leakage of the lysosomal hydrolases into the cytosol leads to lysosomal cell death [62, 63]. Lysosomal cell death can be apoptosis- and necroptosis-independent [68]. We show here that LCL521 rapidly accumulates in the lysosomes. If LCL521 acts as a ceramide analog, it may binds to cathepsin B and D directly to activate cathepsins B and B and cause lysosomal cell death. Although cathepsins are usually considered as downstream executors of lysosomal cell death pathway, they can also initiate lysosomal membrane permeabilization and subsequent lysosomal cell death. This is supported by the observation that cathepsin B deficiency prevents hepatocyte lysosomal membrane permeabilization induced by sphingosine [69]. Therefore, LCL521 may directly activate cathepsins B and D to induce lysosomal cell death. Although ER stress leads to lysosome-mediated protein degradation *via* autophagy [57, 70], the sworn ER and the interrupted autophagy might also be the consequences of lysosomal cell death. This notion also requires further study.

## MATERIALS AND METHODS

### Mice and cells

BALB/c mice were obtained from Charles River Laboratories. Experiments and care/welfare of mice were carried out in accordance with the approved guidelines and according to an approved protocol by the Augusta University Institutional Animal Care and Use Committee. Myeloid J774 (J774A.1) cells were obtained from American Type Culture Collection (Cat# TIB-67, ATCC, Mannassas, VA). Mannassas, VA). ATCC characterizes these cells by morphology, immunology, DNA fingerprint, and cytogenetics. Murine CMS4-met sarcoma cells were generated as previously described [71]. All experimental procedures were performed according to guidelines of Augusta University institutional biosafety committee.

### LCL521 therapy

CMS4-met cells (2.5x10^5^ cells/mouse) were injected into the right flank of BALB/c mice subcutaneously. Thirty days after tumor cell injection, tumor-bearing mice were twice treated with LCL521 (75 mg/kg body weight) with a two day interval between treatments. Tumor tissues were collected and digested in collagenase solution and cell digests were passed through a 75-μM cell strainer. The single cell suspension was stained with anti-CD11b and anti-Gr1 mAbs (Biolegend, San Diego, CA) and analyzed by flow cytometry. Cells were also prepared from spleen, blood, and bone marrow of tumor-free and tumor-bearing mice, stained with anti-CD11b and anti-Gr1 mAbs and analyzed by flow cytometry.

### MDSC functional analysis

Single cell suspension was prepared from spleens of BALB/c mice and passed through 100 μM cell strainers. CD3^+^ T cells were then isolated from the spleen cells using MojoSort Mouse CD3 T Cell Isolation Kit (Biolegend). MDSCs were purified from spleens of tumor-bearing BALB/c mice using CD11b MicroBeads and MACS separation column according to the manufacturer's instructions (Miltenyi Biotec Inc. San Diego, CA). Purities of the isolated T cells were determined by staining the purified cells with anti-CD3 mAb and flow cytometry analysis as previously described [72]. Purities of isolated MDSCs were determined by staining the purified cells with anti-CD11b and anti-Gr1 mAbs and flow cytometry analysis. The purified CD3^+^ T cells were labeled with CellTrace CFSE Cell Proliferation Kit according to the manufacturer's instructions (Life Technologies, Carlsbad, CA), and cultured in anti-CD3 and anti-CD28 mAbs-coated 96-well plates (1.5x10^3^ cells/well). J774M cells were added to the T cell culture at various cell densities. The purified MDSCs from tumor-bearing mice were added to the T cell culture at a cell density of 1x10^6^ cells/well. The cell culture mixtures were collected 3 days later and stained with anti-CD11b and anti-Gr1 mAb, and analyzed by flow cytometry. The CD11b^-^Gr1^-^ cells were gated out and analyzed for CFSE intensity.

### Reagents

LCL521 is lysosomotropic inhibitor of acid ceramidase [65]. LCL521 was synthesized by Lipidomics Shared Resources at Medical University of South Carolina. Z-VAD was obtained from Enzo Life Sciences (Farmingdale, NY). Chloroquine (CQ) was obtained from Sigma-Aldrich, 3-methyladenine (3MA) was obtained from Invivogen (San Diego, CA). FasL (Mega-Fas Ligand, kindly provided by Drs. Steven Butcher and Lars Damstrup at Topotarget A/S, Denmark) is a recombinant fusion protein that consists of three human FasL extracellular domains linked to a protein backbone comprising the dimmer-forming collagen domain of human adiponectin. The Mega-Fas Ligand was produced as a glycoprotein in mammalian cells using Good Manufacturing Practice compliant process in Topotarget A/S (Copenhagen, Denmark).

### Western blotting analysis

Western blotting analysis was performed as previously described [73]. Sources of antibodies are: BAX, BNIP3, Mcl-1 and acid ceramidase; Bcl-xL, Bcl-2, Beclin-1 and Cytochrome C: BD Biosciences (San Diego, CA); BAK, BOK, BIM, BAD, PUMA, xIAP and Cathepsin D: Cell Signaling Tech (Danvers, MA); Cathepsin B: Abcam (Cambridge, MA); β-actin: Sigma-Aldrich (St Luis, MO).

### Cell viability and death analysis

For cell viability assay, cells were seeded in 96-well plates in the absence and presence of various compounds and cultured for approximately 24h. Cell viability was then analyzed by the MTT cell proliferation kit according to the manufacturer's instructions (ATCC, Manassas, VA). Cell death was analyzed as previously described [35]. Briefly, both floating and adherent cells were collected and incubated with propidium iodide (PI) solution and analyzed by flow cytometry. The percentage of cell death was calculated by the formula: % cell death = % PI^+^ in the treatment group - % PI^+^ in the control group as previously described [74].

### Isolation of mitochondria and lysosomes

Isolation of the mitochondria was performed using the Cell Fractionation kit according to the manufacturer's instructions (Abcam). Isolation of the lysosomes was performed using the Lysosome Enrichment kit according to the manufacturer's instructions (Thermo-Fisher Scientific).

### Measurement of sphingolipids

Endogenous sphingolipids (ceramide, sphingosine, and sphingosine 1-phosphate) and level of LCL521 were measured by Lipidomics Shared Resources, Medical University of South Carolina using high-performance liquid chromatography-mass spectrometry approach (LC-MS/MS). Sphingolipids and LCL521 levels were normalized to the total cellular protein contents.

### Cell surface protein analysis

Cells were stained with anti-mouse Fas (BD biosciences), anti-CD11b and anti-Gr1 (Biolegend) mAbs. Isotype-matched control IgG (Biolegend) was used as a negative control. The stained cells were analyzed by flow cytometry as previously described [27].

### Ultrastructure analysis of cellular organelles

Cells were fixed in 4% paraformaldehyde, 2% glutaraldehyde in 0.1 M sodium cacodylate (NaCac) buffer, pH 7.4, postfixed in 2% osmium tetroxide in NaCac, stained en bloc with 2% uranyl acetate, dehydrated with a graded ethanol series and embedded in Epon-Araldite resin. Thin sections were cut with a diamond knife on a Leica EM UC6 ultramicrotome (Leica Microsystems, Inc, Bannockburn, IL), collected on copper grids and stained with uranyl acetate and lead citrate. Cells were observed in a JEM 1230 transmission electron microscope (JEOL USA Inc., Peabody, MA) at 110 kV and imaged with an UltraScan 4000 CCD camera & First Light Digital Camera Controller (Gatan Inc., Pleasanton, CA).

### Quantitative analysis of GFP-LC3 level

J774M cells were transfected with a LC3-GFP-expressing vector by electroporation using the gene Pulser Xcell PC module (Bio-Rad Laboratories). Cells were then treated with LCL521 and directly observed with an inverted fluorescence microscope (Nikon Eclipse TE300). The number of GFP-LC3 dots in each cell was counted in a blind fashion.

### Statistic analysis

Where indicated, data were represented as the mean ± SD. Statistical analysis was performed using two-sided *t* test, with *p*-values<0.05 considered statistically significant.
